# Blood transfusion practices in the ICU of a level 1 trauma centre and tertiary cardiac unit

**DOI:** 10.1186/cc12309

**Published:** 2013-03-19

**Authors:** Y Mustafa, B Pouchet

**Affiliations:** 1 University Hospitals Birmingham NHS Foundation Trust, Birmingham, UK

## Introduction

Blood transfusions are associated with longer ICU and hospital inpatient durations, and an increase in mortality [1]. This study was undertaken to investigate whether the practice of packed red cell (PRC) transfusions in the ICU was in accordance with the best clinical evidence. A number of studies, most notably the TRICC study [2], have shown that indications for ICU blood transfusions are a haemoglobin (Hb) level of <7 g/dl or evidence of acute haemorrhage [3]. These criteria were therefore employed.

## Methods

This study prospectively examined episodes of PRC unit transfusions over a 2-month period in the ICU of a large level 1 trauma centre and a tertiary cardiac unit. The number of PRC units transfused in each episode was recorded by nurses, along with the proposed indication and concurrent Hb level. The data were analysed to assess the number of transfusions administered contrary to the guidelines, along with the average Hb level at which a PRC unit was transfused and the average number of units administered per episode.

## Results

A total 175 units of PRC were transfused in the ICU, over 105 episodes during the 2-month period (excluding immediately postoperative transfusions). Ninety-four units (53.7%) administered in 64 transfusion episodes (61.0%) occurred contrary to the guidelines. In 89.3% of these cases the recorded reason for transfusion was an apparently low Hb level. The median (IQR (range)) Hb level at which patients were transfused: within guidelines was 6.9 g/dl (6.6 to 7.7 (4.8 to 13.9)); within guidelines, excluding cases of acute blood loss, was 6.6 g/dl (6.5 to 6.8 (5.5 to 6.9)); and outside the guidelines was 7.7 g/dl (7.4 to 8.2 (7.0 to 9.7)). One unit of PRC was transfused in 57 episodes (54.3%), 2 units of PRC were transfused in 36 episodes (34.3%), and 3 to 6 units were transfused in 12 episodes (11.4%), with two-thirds of the latter due to acute haemorrhage.

## Conclusion

Our results indicate a liberal transfusion threshold currently exists in the ICU. Patients are frequently receiving excessive PRC transfusions for Hb levels above the recommended concentration. In the 2-month study period, these were associated with a cost of approximately £12,220. We recommend increased staff awareness of the guidelines to reduce the number of unnecessary transfusions. This would decrease exposure of ICU patients to unnecessary risks of blood transfusion, reduce cost of treatment and help to preserve a valuable resource.
